# Inhibition of membrane RANKL production in osteoblasts mediates curcumin-inhibited osteoclastogenesis

**DOI:** 10.1590/1414-431X2026e15332

**Published:** 2026-07-10

**Authors:** Tingwei Gao, Tie Ke, Zhanhao Xiao, Xi Gao

**Affiliations:** 1 Department of Orthopedic Surgery, Fuzhou Second General Hospital, Fuzhou, Fujian, China; 2 Shengli Clinical Medical College of Fujian Medical University, Fuzhou, Fujian, China; 3 Department of Orthopedics, Fuzhou University Affiliated Provincial Hospital, Fujian Provincial Hospital, Fuzhou, Fujian, China

**Keywords:** Curcumin, RANKL, MMP14, Osteoclast, Osteoblast

## Abstract

Curcumin inhibits osteoclastogenesis and mitigates osteoporosis. Our data demonstrated that curcumin decreases membrane-bound RANKL (mRANKL) levels on osteoblasts. Notably, mRANKL exerts a more prominent role in osteoclastogenesis than its soluble counterpart (sRANKL). This study aimed to explore the role of RANKL membrane localization in curcumin-regulated osteoclastogenesis. We primarily investigated the effect of curcumin on mRANKL expression in osteoblasts *in vitro* and *in vivo*. Using fluorescence-activated cell sorting (FACS) for RANKL and co-culture experiments of osteoclast precursors (OCPs) with osteoblasts, we further analyzed the association between curcumin's indirect inhibition of osteoclast differentiation and mRANKL production. Finally, we explored the involvement of matrix metalloproteinase 14 (MMP14) in curcumin-mediated regulation of mRANKL levels. The results showed that curcumin significantly reduced mRANKL expression in osteoblasts. Curcumin also abrogated the upregulated RANKL levels in osteoblasts of Tg-hRANKL transgenic mice. Co-culture assays revealed that curcumin exerted the weakest inhibitory effect on osteoclast differentiation when OCPs were co-cultured with mRANKL-negative osteoblasts. Additionally, curcumin increased MMP14 expression in osteoblasts and enhanced the MMP14-RANKL interaction. Importantly, silencing MMP14 in osteoblasts reversed curcumin-inhibited mRANKL levels and osteoclast differentiation. Collectively, our findings indicated that curcumin inhibited mRANKL production in osteoblasts, which contributes to its therapeutic effect on osteoclastic osteoporosis.

## Introduction

Osteoporosis is a prevalent and debilitating disease in aging populations, characterized by high morbidity and disability rates, and substantial medical burden, posing severe challenges to public health. Optimizing therapeutic strategies for osteoporosis remains a critical focus in clinical and translational research. Curcumin, a bioactive compound extracted from turmeric (*Curcuma longa*) roots, has been shown to exert potent effects in anti-inflammation, antioxidation, lipid regulation, antiviral activity, anti-infection, antitumorigenesis, anticoagulation, anti-liver fibrosis, and anti-atherosclerosis (1). Importantly, curcumin can prevent bone loss, classifying it as a promising anti-osteoporotic phytomedicine. For instance, curcumin effectively improves lumbar bone mineral density (BMD) in ovariectomized (OVX) rats (2), which was corroborated by recent studies (3-6). The ability of curcumin to suppress osteoclastogenesis is a key determinant of its anti-osteoporotic activity. However, the intrinsic mechanisms underlying curcumin-mediated inhibition of osteoclastogenesis remain incompletely understood, hindering the optimization of curcumin-based therapeutic strategies.

Receptor Activator of Nuclear Factor-κB Ligand (RANKL) is the central inducer of osteoclastogenesis. By binding to its receptor RANK, RANKL activates downstream signaling cascades that drive the differentiation and maturation of osteoclasts. RANKL exists in both soluble (sRANKL) and membrane-bound (mRANKL) forms. Endogenously, RANKL is a type II single-pass transmembrane protein that non-covalently assembles into homotrimers (7). Membrane-anchored metalloproteinases, including MMP14, cleave RANKL between its TNF domain and transmembrane region, releasing sRANKL from the cell surface (8,9). Notably, increased mRANKL levels or decreased sRANKL levels enhance osteoclastogenic activity (8). mRANKL mediates direct cell-cell interactions between osteoblasts and osteoclast precursors (OCPs), thereby promoting osteoclastogenesis (10). Furthermore, studies using transgenic mice with impaired mRANKL cleavage capacity have confirmed that mRANKL exhibits higher potency in inducing osteoclast maturation compared to sRANKL (8,11-13). These mutant mice display normal osteoclastogenesis even in the absence of sRANKL, indicating that mRANKL is the primary driver of osteoclast generation (8,11-13). The superior ability of mRANKL to activate RANK signaling may stem from its pre-crosslinking within the phospholipid bilayer prior to binding RANK (12). Collectively, mRANKL levels serve as a reliable indicator of osteoclastic bone loss. Curcumin's anti-osteoclastogenic activity has also been linked to its inhibition of RANKL-induced osteoclastogenesis (6,14-17). Thus, investigating the regulatory effect of curcumin on mRANKL levels holds significant scientific value.

Our experimental data showed that curcumin inhibited mRANKL production on the osteoblast membrane, which may contribute to its protective effect against osteoclast-mediated bone loss. This study aimed to elucidate the underlying mechanism by which curcumin regulates mRANKL production.

## Material and Methods

### Cell line, culture, and curcumin administration

Murine MC3T3-E1 osteoblast precursor cell line was purchased from American Tissue Culture Collection (ATCC). Cells were maintained in DMEM containing 10% fetal bovine serum (FBS) in a humidified environment of 37°C and 5% CO_2_. MC3T3-E1 cell line was tested and authenticated by STR analysis, and all experiments were performed with mycoplasma-free cells. Curcumin used in all experiments was purchased from Sigma-Aldrich (USA). The concentrations, doses, route, and frequency of curcumin *in vitro* and *in vivo* are based on previous studies and identified by pre-experiments (6). All mice were fed the control diet (SPF mouse chow; Shanghai SLAC Laboratory Animal Co., Ltd., China) or 200 mg/kg a day curcumin with the diet for 4 weeks.

### Extraction and induction of OCPs

The tibiae from 4-week-old C57BL/6J mice (Gem Pharmatech, China) were flushed with αlfa-MEM without FBS. Bone marrow cells were incubated in complete αlfa-MEM (+ 10% FBS + 100 U/mL penicillin + 100 μg/mL streptomycin) for 24 h. Non-adherent cells were cultured on dishes with M-CSF (30 ng/mL) for 3 days. The harvested adherent cells are bone marrow-derived macrophages (BMMs), which can be termed as OCPs and induced into mature osteoclasts (18). OCPs were cultured in a humidified environment of 37°C and 5% CO_2_. All OCPs in this study were cocultured with MC3T3-E1 cells to induce mature osteoclasts.

### Coculture of MC3T3-E1 cells and OCPs, and osteoclast differentiation assays

For the coculture, MC3T3-E1 cells were seeded in 48-well plates (4×10^4^ cells/mL). After 24 h of incubation, OCPs (4×10^4^ cells/mL) were seeded on MC3T3-E1 cells and treated with M-CSF (30 ng/mL) for 4 days to induce the mature osteoclasts. Osteoclast differentiation was confirmed by TRAP staining using the relevant kit (Sigma-Aldrich) according to the manufacturer's protocols. TRAP-positive multinucleate cells (over 3 nuclei) were regarded as mature osteoclasts. TRAP-positive cells with ≥5 nuclei were regarded as large osteoclasts.

### Lentiviral transduction

Recombinant lentiviruses encoding shRNA against MMP14 were constructed by homologous recombination between the expression vector (pEX-Puro-Lv105) and shRNA in 293 cells using the lentivirus construction kit according to the manufacturer's protocols. The same method was applied to construct and package the corresponding control vector. After 2 days, supernatants were collected, and MC3T3-E1 cells were incubated in complete DMEM containing lentiviruses and 5 μg/mL polybrene at a multiplicity of infection (MOI) of 40 for 2 d. The infected cells were selected using puromycin (10 μg/mL). The transduction efficiency of the viral gene was detected using western blot analysis.

### Western blot analyses

The cell lysate from indicated cells was prepared, packaged into 10% SDS-PAGE gel and transferred to polyvinylidene fluoride membrane (PVDF). Then, the PVDFs were combined with antibodies targeting RANKL (1:500; Santa Cruz Biotechnology, Inc., USA) and MMP14 (1:2000; Abcam, UK). Horseradish peroxidase (HRP)-linked secondary antibody was used as the secondary antibody. The signals were observed using artificial exposure.

### Coimmunoprecipitation (Co-IP) assays

The total cellular protein was extracted by RIPA Lysis and Extraction Buffer (ThermoFisher Scientific, USA). Next, we rinsed the beads using 100 μL of iced buffers, added 100 μL of antibody-binding buffer to revolve the antibody and magnetic beads for 30 min, and then rinsed the beads 3 times using 200 μL of buffers for 5 min each time. The lysates and antibody-bound magnetic beads were incubated for 1 h at room temperature and washed using 200 μL of buffers for 5 min each time. A total of 20 μL of eluent was used to rinse the beads once and the supernatant was removed. The lysates were extracted for Co-IP with anti-RANKL antibody, and subsequently, precipitates were examined using Western blot with anti-MMP14 antibody.

### Quantitative real-time PCR (qRT-PCR) analyses

The total RNA was extracted and purified by the TRIzol method. Synthesis of cDNA and real-time quantitative PCR (qRT-PCR) measurements were carried out as described previously (19). The designed primer sequences for qRT-PCR were: cathepsin K (CTSK): 5′-GGAAGAAGACTCACCAGAAGC-3′ (forward) and 5′-GTCATATAGCCGCCTCCACAG-3′ (reverse); matrix metalloproteinase 9 (MMP9): 5′-CCTGTGTGTTCCCGTTCATCT-3′ (forward) and 5′-ACCCGAATCTAGTAAGGTCGC-3′ (reverse); TRAP: 5′-GCTGGAAACCATGATCACCT-3′ (forward) and 5′-TTGAGCCAGGACAGCTGAGT-3′ (reverse); cyclophillin A: 5′-CGAGCTCTGAGCACTGGAGA-3′ (forward) and 5′-TGGCGTGTAAAGTCACCACC-3′ (reverse).

In the qRT-PCR detection, the melting curve was analyzed to ensure that there was no amplification artifact. qRT-PCR was carried out using SYBR Premix Ex TaqTM kit (TaKaRa, Japan) and ABI7500 PCR system (Applied Biosystems, ThermoFisher Scientific).

### Animal experiments

After breeding, 12-week-old male Tg-hRANKL mice and littermate wild-type mice were used in this experiment (C57BL/6J, n=6/group). All mice were housed in a specific pathogen-free facility with barrier in a room temperature of 20∼30°C and humidity of 60∼80% and were fed the SPF mouse chow (Shanghai SLAC Laboratory Animal Co., Ltd.) and sterile water. Tg-hRANKL mice were assigned to two experimental groups by numbering, ensuring the same number of mice in both groups. After euthanasia, the tibiae and femurs were collected, wrapped in the gauze soaked in 0.9% saline, and stored at -20°C. The researchers on our team simultaneously gavaged two mice to minimize interference with the treatment order. Then, the researchers simultaneously sacrificed the mice and extracted bone tissue and peripheral blood. The group allocation at the different stages of the experiment was performed by laboratory staff who were not authors of the study. All experimental protocols were approved by the Institutional Animal Care Review Committee of Fujian Provincial Hospital (approval number: IACUC-FPH-PZ-20251229[039]). The Canadian Council on Animal Care guidelines (CCAC) were followed during the course of the experiment. All mice were anesthetized with isoflurane (2%, inhalation anesthesia), and then sacrificed via cervical dislocation. The mice were considered dead when the heart and breathing stopped.

### Micro-computed tomography (micro-CT) analyses

Bruker Micro-CT Skyscan 1276 system (Kontich, Belgium) was used for three-dimensional (3D) analysis of the cancellous bones in the distal femur metaphysis. The scanning settings were as following: voxel size of 6.533481 μm, medium resolution of 55 kV, 200 mA, 1 mm Al filter, and integration time of 384 ms. BMD measurements were calibrated according to the manufacturer's calcium hydroxyapatite (CaHA) model. The analysis was carried out using the manufacturer's evaluation software. Three dimensional (3D) reconstruction was completed by NRecon (version 1.7.4.2; Micro Photonics). The parameters consisted of BMD, bone volume/tissue volume (BV/TV), trabecular thickness (Tb.Th), trabecular number (Tb.N), bone surface/bone volume (BS/BV), trabecular separation (Tb.Sp), and cortical thickness (Ct.Th) (n=6, per group).

### Hematoxylin and eosin (H&E) staining and TRAP staining in bone tissues

The indicated tibiae from all mice were fixed in 4% paraformaldehyde (PFA) for 48 h and decalcified in 10% EDTA (pH 7.3) for 2 weeks at 4°C. Next, all specimens were dehydrated in graded ethanol series and embedded in paraffin. All sections (5-μm thick) were stained with H&E staining kit (n=6, per group). Image-Pro Plus (IPP, version 7.0, USA) software was used to analyze trabecular bone area (%Tb.Ar) of the H&E-stained sections. Subsequently, the above sections were stained with TRAP staining kit according to the standard protocols, and the eyepiece grid was applied to detect the number of osteoclasts in the stained sections.

### Immunofluorescent staining in bone tissues

For immunofluorescence staining *in vivo* (RANKL, 1:300; Runx2, 1:500; Santa Cruz Biotechnology), the sections were incubated in citrate buffer (10 mM citric acid, pH 6.0) overnight at 60°C to unmask the antigens. We incubated the primary antibodies overnight at 4°C and the secondary antibodies for 1 h at room temperature (n=6, per group). Finally, the nuclei were counterstained with DAPI. The intensity of single fluorescence and overlapped fluorescence was analyzed by Image-Pro Plus (IPP), and the number of cells expressing the corresponding fluorescence was observed and counted.

### Flow cytometry analysis of RANKL's membrane expression

After blockage of nonspecific binding with rat anti-mouse CD16/32 monoclonal antibody (TruStain FcX™ PLUS, BioLegend, USA), each group of treated MC3T3-E1 cells or primary osteoblasts was stained using Biotin anti-mouse CD254 (RANKL) antibody according to the manufacturer's protocols. Next, a flow cytometer (BD Accuri C6 Plus, BD Biosciences, USA) was applied to evaluate and quantitatively analyze the percentage of RANKL-positive cells.

### FACS of MC3T3-E1 cells

After blockage of nonspecific binding with rat anti-mouse CD16/32 monoclonal antibody, Biotin anti-mouse CD254 (RANKL) antibody was used to stain MC3T3-E1 cells. Next, different clusters of cells were collected on MoFlo XDP flow cytometer (Beckman Coulter, USA). The collected RANKL^+^ and RANKL^-^ cells were cultured in preparation for subsequent experiments.

### ELISA analyses

Serum concentrations of RANKL and C-terminal cross-linking telopeptide (CTX) were detected using ELISA kits (Signalway Antibody, USA). The assays were performed according to the manufacturer's protocols.

### Statistical analyses

All data are reported as means±SE. Statistical analyses were performed using one-way ANOVA or Student's *t*-test. Tukey's test was used for *post hoc* multiple comparisons of one-way ANOVA. The threshold for the P-value was set to 0.05. One sample *t*-test was used to assess whether the data met the assumptions of the statistical approach. All statistical analyses were performed using SPSS 19.0 software (IBM, USA).

## Results

### Curcumin inhibited membrane RANKL levels in osteoblasts

**Figure 1:**
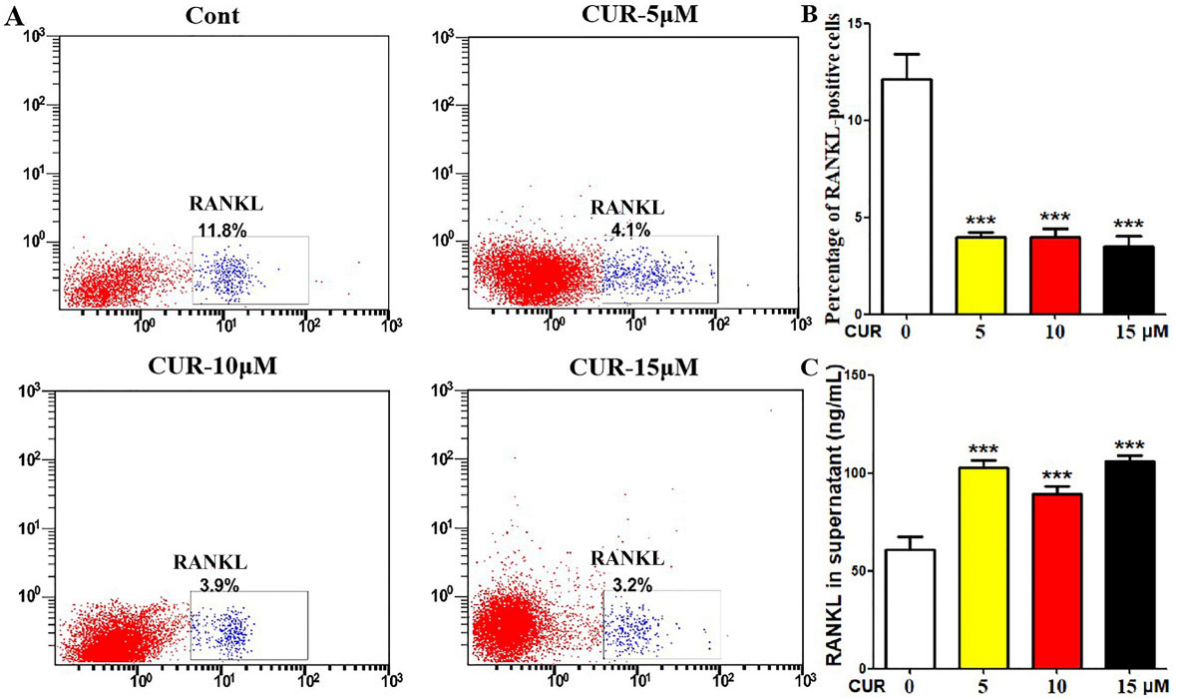
Curcumin inhibited membrane RANKL levels in osteoblasts. **A** and **B**, MC3T3-E1 cells were treated with curcumin (0, 5, 10, or 15 μM) for 24 h, and the percentage of RANKL-positive cells was determined by flow cytometry. **C**, sRANKL levels in the culture supernatant were measured by ELISA after 24-h curcumin treatment (0, 5, 10, or 15 μM). Experiments were repeated at least three times. Data are reported as means±SE. ***P<0.001 compared to Control (ANOVA). Cont: control (no curcumin); CUR: curcumin.

We first evaluated the effect of curcumin on mRANKL expression in MC3T3-E1 osteoblasts. Flow cytometry analysis of RANKL revealed that curcumin inhibited membrane RANKL levels in MC3T3-E1 cells at all tested concentrations (Figure 1A and B). Since flow cytometry without cell permeabilization specifically detects membrane-bound proteins, these results confirmed curcumin's inhibitory effect on mRANKL in osteoblasts. Consistent with this, ELISA assays showed that curcumin increased sRANKL production in the culture supernatant at all tested concentrations (Figure 1C).

### Curcumin alleviated bone loss in Tg-hRANKL mice

**Figure 2:**
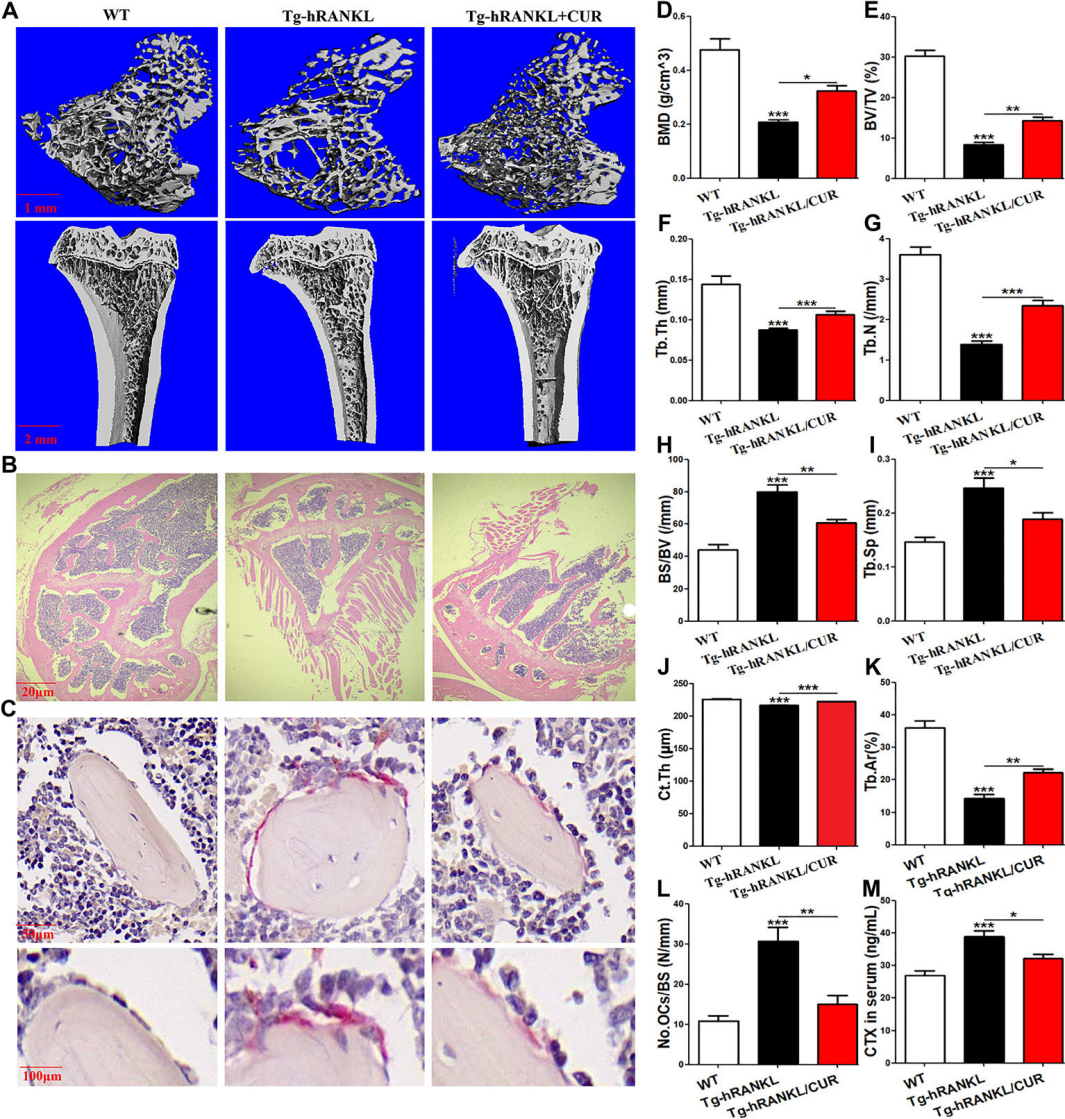
Curcumin alleviated bone loss in Tg-hRANKL mice. **A**, Representative 3D micro-CT reconstructed images of femurs from Tg-hRANKL mice fed a control diet or a diet supplemented with 200 mg/kg per day curcumin for 4 weeks (n=6/group), and wild-type (WT) littermate controls, showing bone mass and microstructure. Scale bars, 1 mm and 2 mm. **B**, Representative H&E-stained tibial sections from each group. Scale bar, 20 μm. **C**, Representative TRAP-stained tibial sections from each group. Scale bars, 50 μm and 100 μm. **D**-**J**, Trabecular bone parameters (BMD, BV/TV, BS/BV, Tb.Th, Tb.N, Tb.Sp, and Ct.Th) were analyzed by micro-CT. **K**, Trabecular area (Tb.Ar) was quantified by H&E staining and IPP software. **L,** Osteoclast abundance was detected and quantified by TRAP staining and the eyepiece grid. **M**, Serum levels of CTX were measured by ELISA. Data are reported as means±SE. *P<0.05; **P<0.01; ***P<0.001 (ANOVA). WT: wild-type littermate controls; CUR: curcumin; BMD: bone mineral density; BV/TV: bone volume/tissue volume; BS/BV: bone surface/bone volume; Tb.Th: trabecular thickness; Tb.N: trabecular number; Tb.Sp: trabecular separation; Ct.Th: cortical thickness.

To verify the *in vivo* effect of curcumin on mRANKL levels, we used Tg-hRANKL transgenic mice, which overexpress human RANKL. Micro-CT 3D reconstruction demonstrated that curcumin partially restored reduced bone mass and impaired bone microarchitecture in Tg-hRANKL mice (Figure 2A). Quantitative micro-CT analysis showed that curcumin reversed the decreases in BMD, BV/TV, Tb.Th, Tb.N, and Ct.Th, as well as the increases in /BV and Tb.Sp, in Tg-hRANKL mice (Figure 2D-J). H&E staining revealed that curcumin mitigated the bone loss phenotype in Tg-hRANKL mice, including thinner growth plates, reduced and disorganized trabecular bone, expanded bone marrow cavities, and increased marrow adiposity (Figure 2B). Quantitative analysis confirmed that curcumin partially rescued the reduced trabecular area in Tg-hRANKL mice (Figure 2K). Importantly, TRAP staining and ELISA assays showed that curcumin reversed the increased osteoclast abundance and elevated serum levels of osteoclastic marker CTX in Tg-hRANKL mice (Figure 2C, L, and M). These results demonstrated that curcumin suppressed RANKL-induced bone loss and osteoclast activity *in vivo*, validating our experimental model.

### Curcumin abrogated elevated RANKL levels in osteoblasts of Tg-hRANKL mice

**Figure 3:**
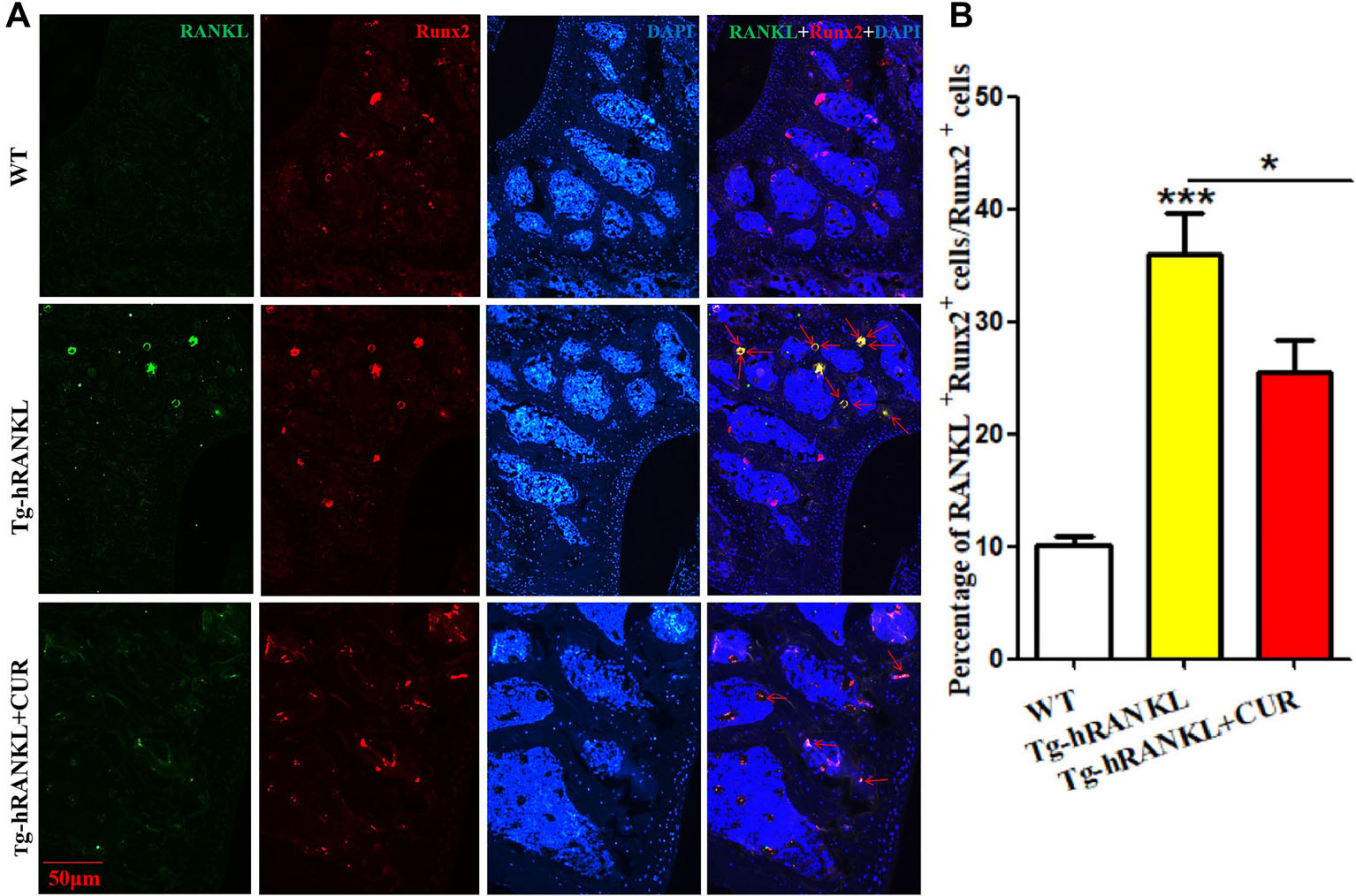
Curcumin abrogated elevated RANKL levels in osteoblasts of Tg-hRANKL mice. **A**, Representative immunofluorescence images of RANKL and Runx2 in bone tissues from each group (single and merged channels; n=6/group). Yellow fluorescence indicates colocalization of RANKL and Runx2. Scale bar, 50 μm. **B**, The histogram shows the quantitative ratio of RANKL^+^Runx2^+^ cells to total Runx2^+^ cells (percentage). Data are reported as means±SE from 6 independent samples. *P<0.05; ***P<0.001 (ANOVA). WT: wild-type littermate controls; CUR: curcumin.

Using Tg-hRANKL mice, we further explored the *in vivo* effect of curcumin on mRANKL expression in osteoblasts. Immunofluorescence staining showed that RANKL fluorescence intensity was significantly higher in osteoblasts (identified by the marker Runx2) of Tg-hRANKL mice, and this elevation was effectively suppressed by curcumin treatment (Figure 3A and B). Combined with the results in Figure 2, these data indicated that reduced mRANKL production in osteoblasts contributed to curcumin's *in vivo* protective effects.

### Curcumin minimally inhibited osteoclast differentiation when co-cultured with mRANKL-negative osteoblasts

**Figure 4:**
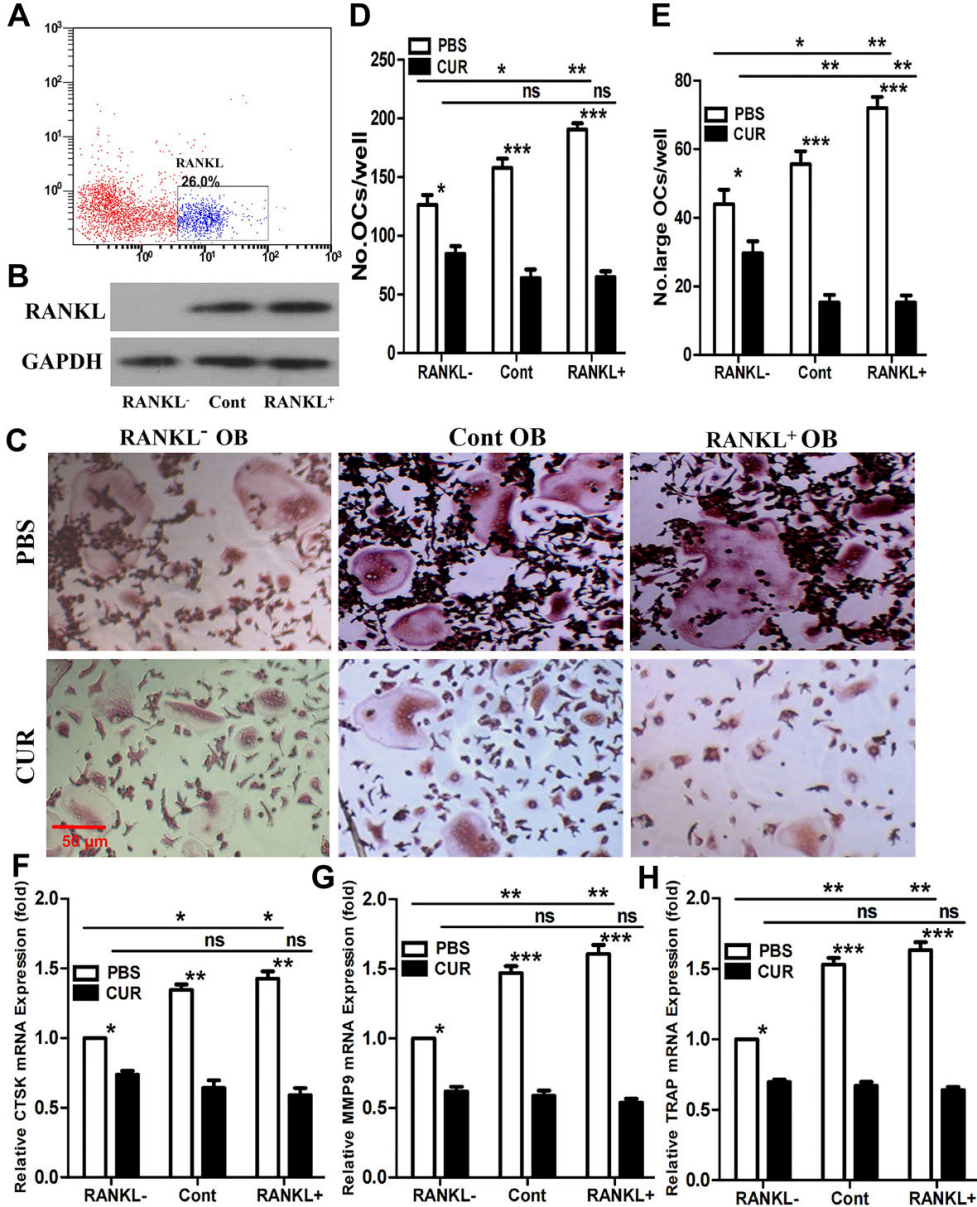
Curcumin minimally inhibited osteoclast differentiation when co-cultured with mRANKL-negative osteoblasts (OB). **A**, FACS plots showing cell sorting efficiency and the percentage of RANKL-positive and RANKL-negative fractions. **B**, RANKL protein levels in sorted MC3T3-E1 cells were confirmed by western blot. **C**, Enriched MC3T3-E1 cells were incubated for 24 h, followed by seeding with osteoclast precursors (OCPs) and treatment with M-CSF (30 ng/mL) for 4 days in the presence of curcumin (10 μM) or saline (PBS). Mature osteoclasts were identified as TRAP-positive multinucleated cells. Scale bar, 50 μm. **D**, Quantitative analysis of mature osteoclasts in (**C**). **E**, Quantitative analysis of large osteoclasts (≥5 nuclei) in (**C**). **F**-**H**, mRNA expression levels of *CTSK*, *MMP9*, and *TRAP* were measured by qRT-PCR after treatment as described in (**C**). Experiments were repeated at least three times. Data are reported as means±SE. *P<0.05; **P<0.01; ***P<0.001 (ANOVA); ns: not significant. Cont: unsorted control; CUR: curcumin; OB: osteoblasts.

To clarify the significance of mRANKL in curcumin's indirect inhibition of osteoclastogenesis, we sorted RANKL-positive (26%) and RANKL-negative (74%) MC3T3-E1 cells by FACS (Figure 4A). Western blot analysis confirmed the sorting efficiency (Figure 4B). We then cocultured OCPs with sorted osteoblasts in the presence or absence of curcumin to evaluate osteoclastogenic capacity. As shown in Figure 4C-E, OCPs cocultured with RANKL-negative MC3T3-E1 cells formed significantly fewer and smaller mature osteoclasts. Curcumin strongly inhibited the number and size of mature osteoclasts in co-cultures with RANKL-positive or unsorted control osteoblasts (osteoclast size was defined as TRAP-positive cells with ≥5 nuclei). However, curcumin exerted only a minimal inhibitory effect on osteoclast number and size in the RANKL-negative group (Figure 4C-E). Statistical analysis confirmed that the inhibitory effect of curcumin on osteoclast differentiation and size was significantly weaker in the RANKL-negative group compared to the RANKL-positive and control groups (Table 1). No significant difference in curcumin's inhibitory efficacy was observed between the RANKL-positive and control groups (Table 1). Consistent with these findings, the expression of osteoclast-specific genes (*CTSK*, *MMP9*, and *TRAP*) showed similar trends (Figure 4F-H). Notably, after curcumin treatment, there was no significant difference in osteoclast number and gene expression levels between each group, and size in the RANKL-negative group was greater than that in the other two groups (Figure 4C-H), indicating that curcumin effectively inhibited osteoclast formation, but had limited effects on osteoclast fusion in the absence of mRANKL.

**Table 1:** Comparison of differences in the number and size of mature osteoclasts (OCs) from each group after curcumin treatment (ANOVA).

Group comparison	ΔOCs number(P value)	ΔLarge OCs number(P value)
RANKL-positive sorting *vs* Control	0.057	0.100
RANKL-positive sorting *vs* RANKL-negative sorting	0.001	0.002
Control *vs* RANKL-negative sorting	0.006	0.021

### Curcumin upregulated MMP14 expression in osteoblasts

**Figure 5:**
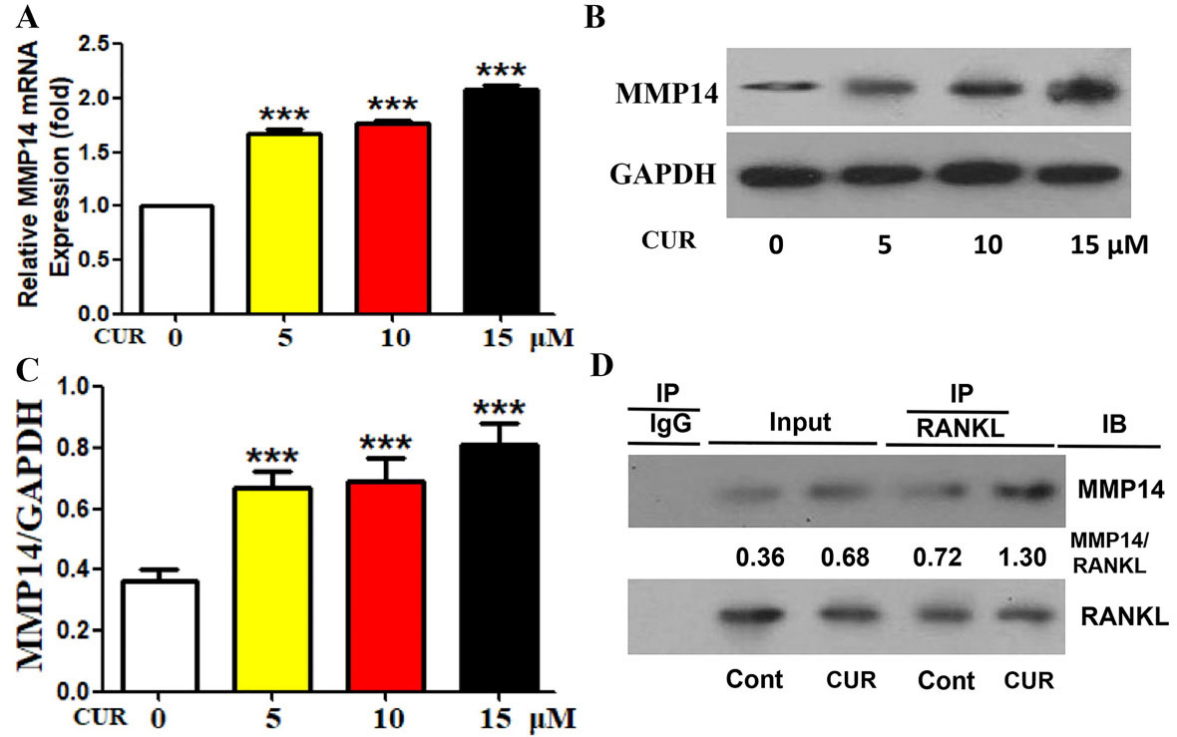
Curcumin upregulated MMP14 expression in osteoblasts. **A**, MMP14 mRNA levels in MC3T3-E1 cells were measured by qRT-PCR after 24-h curcumin treatment (0, 5, 10, or 15 μM). **B** and **C**, MMP14 protein levels in MC3T3-E1 cells were analyzed by western blot after 24-h curcumin treatment (0, 5, 10, or 15 μM). **D**, MC3T3-E1 cell lysates were immunoprecipitated with anti-RANKL antibody, and the precipitate was detected by western blot using anti-MMP14 antibody. Experiments were repeated at least three times. Data are reported as means±SE. ***P<0.001 (ANOVA). Cont: control (no curcumin); CUR: curcumin.

sRANKL is generated by cleavage of mRANKL by sheddases, with MMP14 being a key mediator of RANKL shedding (8). MMP14 has been shown to exhibit strong RANKL-shedding activity (8,13), making it a critical target for curcumin-mediated regulation of mRANKL levels. We first examined the effect of curcumin on MMP14 expression in MC3T3-E1 cells. qRT-PCR and western blot analyses showed that curcumin increased MMP14 mRNA and protein levels in a concentration-dependent manner (Figure 5A-C). Co-immunoprecipitation assays further demonstrated that curcumin enhanced the interaction between RANKL and MMP14 in MC3T3-E1 cells (Figure 5D). These results suggested that curcumin modulated RANKL shedding via MMP14 in osteoblasts.

### MMP14 silencing reversed curcumin-induced inhibition of membrane RANKL levels

**Figure 6:**
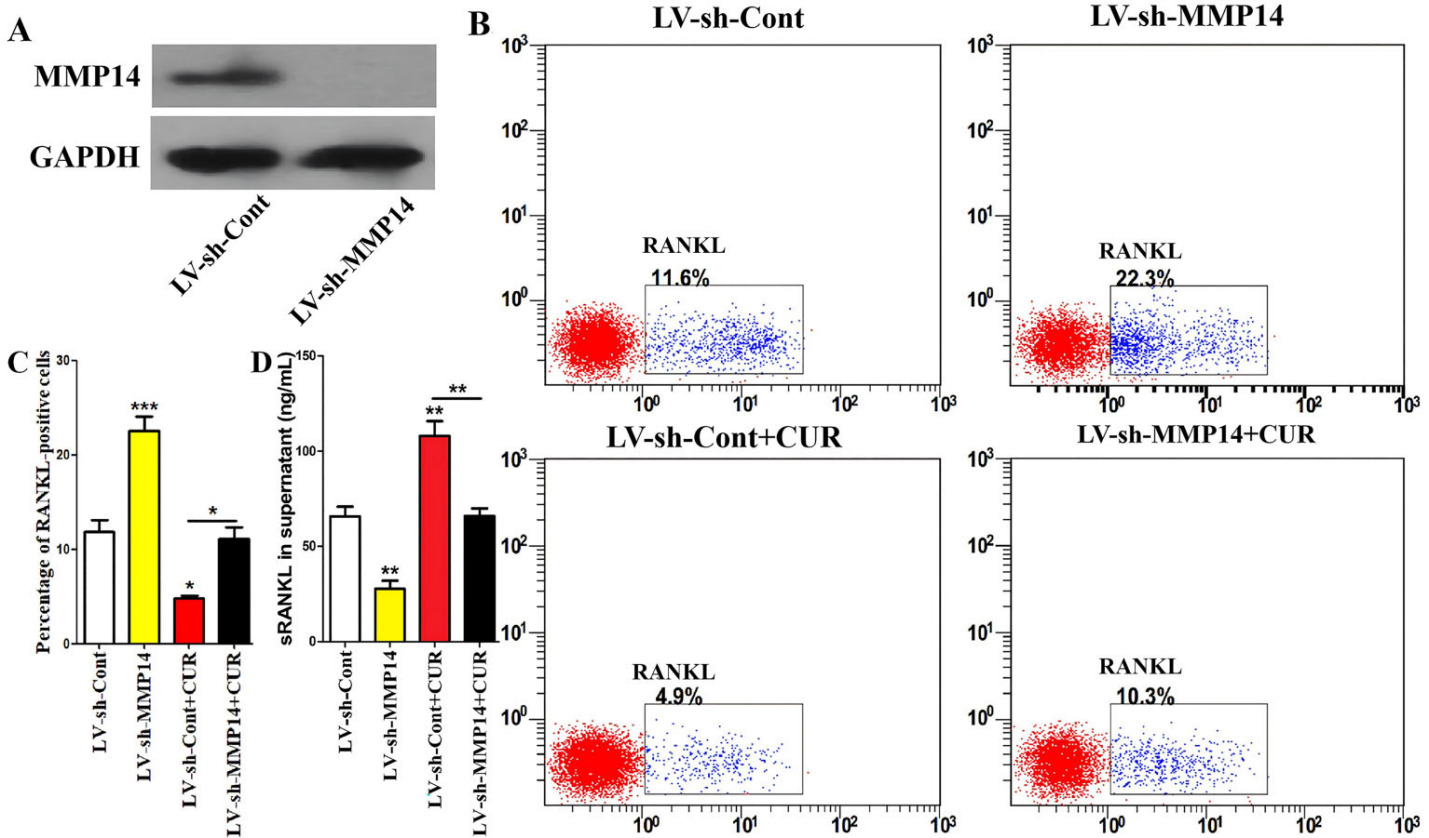
MMP14 silencing reversed curcumin-induced inhibition of membrane RANKL levels. **A**, MMP14 silencing efficiency in MC3T3-E1 cells was confirmed by western blot 24 h after lentiviral transduction. **B** and **C**, Lentivirus-transduced cells were treated with curcumin (10 μM) for 24 h, and the percentage of RANKL-positive cells was determined by flow cytometry. **D**, sRANKL levels in the supernatant of lentivirus-transduced cells were measured by ELISA after 24-h curcumin treatment (10 μM). Experiments were repeated at least three times. Data are reported as means±SE. *P<0.05, **P<0.01, ***P<0.001 (ANOVA). CUR: curcumin; LV: lentivirus; sh: shRNA.

To confirm the role of MMP14 in curcumin-mediated regulation of mRANKL, we silenced MMP14 in MC3T3-E1 cells using RNA interference (RNAi) (Figure 6A). Flow cytometry analysis showed that MMP14 silencing not only increased membrane RANKL levels but also abrogated the inhibitory effect of curcumin on membrane RANKL expression (Figure 6B and C). ELISA assays further confirmed that MMP14 silencing reduced sRANKL levels in the supernatant and reversed curcumin-induced upregulation of sRANKL (Figure 6D). These data indicated that MMP14 is essential for curcumin-mediated inhibition of mRANKL production in osteoblasts.

### MMP14 silencing in osteoblasts reversed curcumin-suppressed osteoclast differentiation

**Figure 7:**
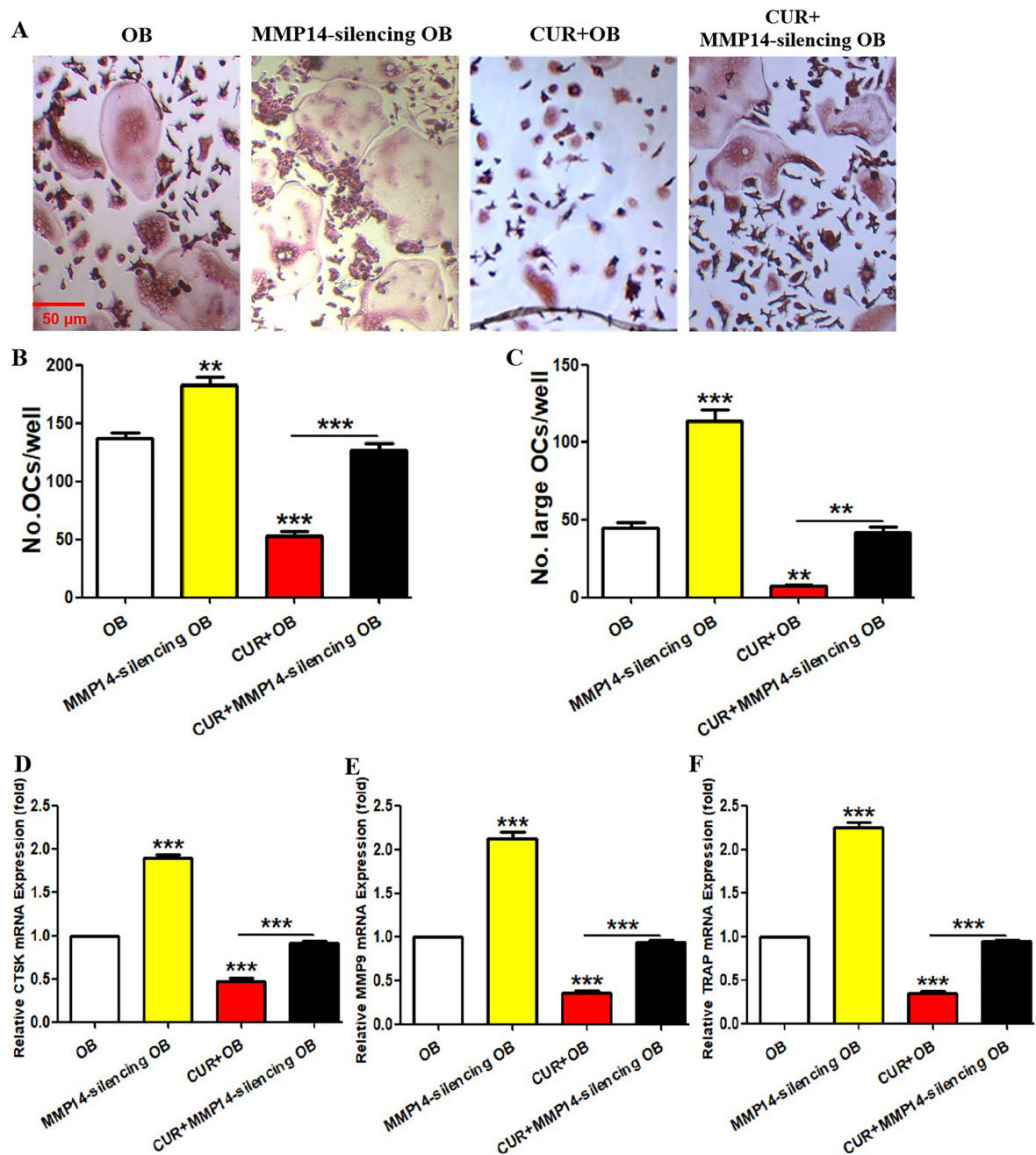
MMP14 silencing in osteoblasts reversed curcumin-suppressed osteoclast differentiation. **A**, MMP14-silenced MC3T3-E1 cells were incubated for 24 h, followed by seeding with osteoclast precursors (OCPs) and treatment with M-CSF (30 ng/mL) for 4 days in the presence or absence of curcumin (10 μM). Mature osteoclasts were identified as TRAP-positive multinucleated cells. Scale bar, 50 μm. **B**, Quantitative analysis of mature osteoclasts in (**A**). **C**, Quantitative analysis of large osteoclasts (≥5 nuclei) in (**A**). **D**-**F**, mRNA expression levels of *CTSK*, *MMP9*, and *TRAP* were measured by qRT-PCR after treatment as described in (**A**). Experiments were repeated at least three times. Data are reported as means±SE. **P<0.01; ***P<0.001 (ANOVA). CUR: curcumin; OB: osteoblasts; OCs: osteoclasts.

Finally, we used a co-culture model to evaluate the effect of MMP14-silenced osteoblasts on curcumin-mediated inhibition of osteoclastogenesis. As shown in Figure 7A-C, MMP14 silencing in MC3T3-E1 cells increased the number and size of mature osteoclasts and reversed the inhibitory effect of curcumin on osteoclast formation and enlargement. Consistent with these observations, the expression of osteoclast-specific genes (*CTSK*, *MMP9*, and *TRAP*) showed corresponding trends (Figure 7D-F). These results confirmed that MMP14 in osteoblasts mediated curcumin's inhibitory effect on osteoclastogenesis.

## Discussion

As a representative anti-osteoclastogenic agent, the pharmacological mechanisms of curcumin require further elucidation. Previous studies have shown that curcumin inhibits RANKL-mediated biological effects during osteoclastogenesis (6,14-17). Given that mRANKL plays a more critical role in osteoclastogenesis and bone loss than sRANKL (8,11-13), we aimed to address a critical scientific question: does curcumin inhibit osteoclastic bone loss by reducing mRANKL production? Using *in vitro* and *in vivo* models, we report for the first time a novel mechanism underlying curcumin's anti-osteoclastogenic activity by targeting RANKL membrane localization.

Our *in vitro* results demonstrated that curcumin inhibited mRANKL levels in osteoblasts in a concentration-dependent manner. Tg-hRANKL mice, which overexpress RANKL, served as an ideal *in vivo* model to evaluate curcumin's efficacy (20,21). Consistent with *in vitro* findings, curcumin alleviated bone loss and suppressed RANKL fluorescence in osteoblasts of Tg-hRANKL mice. Previous studies have focused on curcumin's effects on RANKL signaling in OCPs: Yang et al. showed that curcumin exerts immunomodulatory effects on RANKL-stimulated osteoclast formation (15); curcumin disrupts RANKL signaling in OCPs, reducing nuclear factor of activated T cells 2 (NFAT2) expression and mitogen-activated protein kinase (MAPK) activity (17); and curcumin inhibits osteoclastogenesis by attenuating RANKL-induced autophagy in OCPs (6). Other studies have also reported curcumin's regulatory effects on RANKL-dependent signaling in OCPs (14,16). However, the role of curcumin in modulating RANKL forms (membrane-bound *vs* soluble) has not been previously addressed. Our study fills this gap by confirming that curcumin regulates RANKL localization in osteoblasts, reducing mRANKL levels while increasing sRANKL production. Although both mRANKL and sRANKL can activate OCPs and induce bone resorption, mRANKL-mediated cell-cell interactions provide a more stable signal for osteoclast development. Thus, reduced mRANKL levels impair osteoclast generation, consistent with previous findings that mRANKL is more potent than sRANKL in inducing osteoclast maturation (8,11-13). Our co-culture experiments further confirmed that curcumin's inhibitory effect on osteoclastogenesis was significantly weaker in the presence of RANKL-negative osteoblasts, indicating that curcumin suppresses osteoclastogenesis by downregulating mRANKL in osteoblasts. Notably, no significant difference in curcumin's efficacy was observed between RANKL-positive and control groups, suggesting that RANKL form switching is not the sole mediator of curcumin's effects *in vitro*. Additionally, curcumin's limited effect on osteoclast fusion in the RANKL-negative group also supports that mRANKL production belongs to its effective target.

MMP14 is a key sheddase that cleaves mRANKL to generate sRANKL, making it critical for curcumin-mediated RANKL form switching. Our data showed that curcumin upregulated MMP14 mRNA and protein levels and enhanced the MMP14-RANKL interaction in osteoblasts. Furthermore, MMP14 silencing reversed curcumin-induced inhibition of mRANKL and suppression of osteoclastogenesis. These findings indicated that curcumin promoted mRANKL cleavage by upregulating MMP14, thereby reducing mRANKL levels and inhibiting osteoclast formation. This reveals a novel pathway for curcumin's pharmacological activity, though the molecular mechanism by which curcumin upregulates MMP14 requires further investigation.

**Figure 8:**
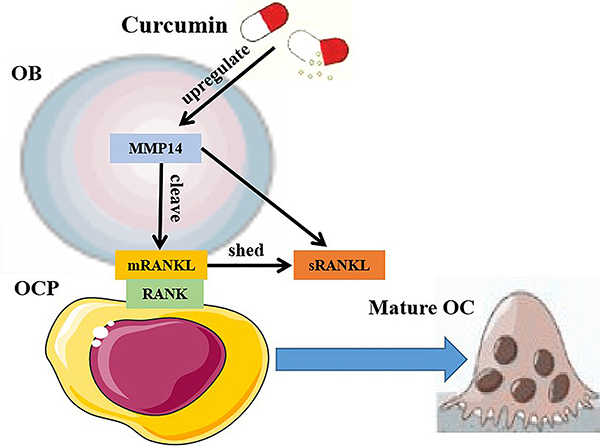
Working model of curcumin's mechanism of action. Compared to sRANKL, direct binding of mRANKL to RANK on osteoclast (OC) precursors (OCPs) more efficiently promotes osteoclast differentiation. Curcumin upregulates MMP14 expression in osteoblasts (OB), enhancing mRANKL cleavage and sRANKL shedding. This mechanism contributes to curcumin-mediated inhibition of osteoclast formation.

A working model of our findings is presented in Figure 8. This study lays the groundwork for identifying curcumin-derived compounds that interact with MMP14, which may enhance curcumin's bioactivity for clinical applications. Osteoprotegerin (OPG), a secreted glycoprotein produced by osteoblasts, acts as a decoy receptor for RANKL, blocking RANKL-RANK binding and inhibiting osteoclastogenesis. While our study focuses on curcumin's regulation of RANKL protein localization, future studies should explore its effects on the OPG-RANKL-RANK axis to fully elucidate curcumin-RANKL relationship.

In conclusion, our research demonstrated for the first time that curcumin inhibited mRANKL production in osteoblasts, which mediates its anti-osteoclastogenic activity. Mechanistically, curcumin enhanced MMP14 expression to promote mRANKL cleavage, thereby reducing osteoclastogenesis. These findings provide new insights into curcumin's anti-osteoporotic effects, facilitating the optimization of curcumin-based therapies and the development of more potent derivatives.

## Data Availability

All data generated or analyzed during this study are included in this published article.
